# Light control of RTK activity: from technology development to translational research

**DOI:** 10.1039/d0sc03570j

**Published:** 2020-09-07

**Authors:** Anna V. Leopold, Vladislav V. Verkhusha

**Affiliations:** a Medicum , Faculty of Medicine , University of Helsinki , Helsinki 00290 , Finland; b Department of Anatomy and Structural Biology and Gruss-Lipper Biophotonics Center , Albert Einstein College of Medicine , Bronx , NY 10461 , USA . Email: vladislav.verkhusha@einsteinmed.org

## Abstract

Inhibition of receptor tyrosine kinases (RTKs) by small molecule inhibitors and monoclonal antibodies is used to treat cancer. Conversely, activation of RTKs with their ligands, including growth factors and insulin, is used to treat diabetes and neurodegeneration. However, conventional therapies that rely on injection of RTK inhibitors or activators do not provide spatiotemporal control over RTK signaling, which results in diminished efficiency and side effects. Recently, a number of optogenetic and optochemical approaches have been developed that allow RTK inhibition or activation in cells and *in vivo* with light. Light irradiation can control RTK signaling non-invasively, in a dosed manner, with high spatio-temporal precision, and without the side effects of conventional treatments. Here we provide an update on the current state of the art of optogenetic and optochemical RTK technologies and the prospects of their use in translational studies and therapy.

## Abstract 2

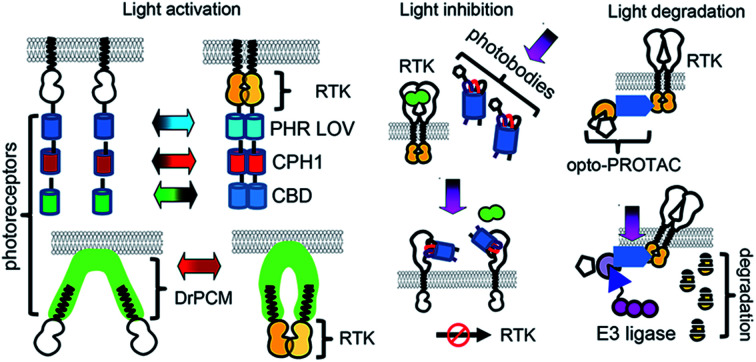
Optogenetical and optochemical approaches can be used to control RTK signalling instead of growth factors, antibodies and small-molecule inhibitors.

## Introduction

Receptor tyrosine kinases (RTKs) are cell surface receptors activated by diverse ligands and controlling cell fate.^1^ Excessive RTK activation leads to oncogenesis whereas insufficient RTK signaling is linked to diabetes mellitus, neurodegeneration, growth delay and improper wound healing.^2^–^4^ Diseases related to RTK activity impose a heavy burden on health-care systems. Inhibition of RTKs with small-molecule inhibitors and monoclonal antibodies (mAbs) is conventional therapy in various cancers.^5^ Activation of RTKs with various ligands (replacement therapy), such as insulin and growth factors (GFs), is used to treat diabetes,^2^ neurodegeneration,^6^ wound healing and muscle regeneration.^7^ While insulin as a hormone acts on multiple organs and tissues,^2^ the activity of other RTK ligands is usually localized and their use for therapeutic purposes should be spatio-temporally controlled.

Conventional therapies of diseases linked to aberrant RTK signaling usually rely on intravenous infusion of RTK ligands, mAbs or small-molecule inhibitors. Intravenous infusion results in the non-targeted action of injected substances on all organs and tissues, frequently leading to complications that vary in severity. For example, suppression of EGFR signaling with therapeutic anti-EGFR mAbs or inhibitors is used in cancer therapy, but EGFR also plays a central role in skin homeostasis and cardiovascular cell survival. As a result, non-discriminative inhibition of EGFR signaling in a whole organism leads to skin rashes and cardiac toxicity.^8^ Similarly, activation of TrkA signaling *via* intracerebral infusion of NGF emerged as a potential therapy for Alzheimer's disease. Clinical trials demonstrated that whereas it slowed disease progression, it also caused back pain due to NGF diffusion into the spinal cord where activation of TrkA leads to secretion of prostaglandins.^6^ To avoid side effects of conventional therapies and to improve their efficacy, a targeted and controlled delivery of GFs and mAbs to their sites of action is required. It can be achieved by engineering of sophisticated delivery vehicles that are reviewed elsewhere.^9^

Recently, two novel technologies to control RTK activity and its downstream signaling with light have been developed. In the first one, optogenetic control of RTK signaling relies on genetically encoded chimeric proteins, called opto-RTKs, which are engineered to comprise photoreceptors fused to intracellular RTK domains.^10^–^12^ These include dimerizing opto-RTKs based on various photoreceptors^10^,^11^,^13^ and RTK oligomerizing techniques, such as “clustering indirectly using cryptochrome 2” (CLICR).^14^ In the second one, RTK is activated optochemically using semi-genetically encoded RTK chimeras in which dimerization or conformational changes are put under the control of photocaged small molecules.^15^,^16^ Other optochemical techniques include photocaging of amino acid residues in the kinase domain^17^ and photocaging of RTK activators like DNA aptamers,^7^ RTK inhibition with light-activatable anti-RTK antibodies (photobodies)^18^,^19^ and RTK degradation with an opto-PROTAC (proteolysis targeting chimera) technique.^20^

Here we first describe the principles of design and the major characteristics of modern optogenetic and optochemical tools to optically manipulate RTK functions and RTK downstream signaling. We then discuss how inhibition or destruction of endogenous RTKs with light could be used in cancer therapy and how opto-RTKs and optochemical means of controlling endogenous RTKs could be used to treat insufficient RTK signaling. We next discuss current challenges and possible ways to overcome them for opto-RTK implementation in translational research and therapy. Lastly, we provide an outlook on the future development of optogenetic and optochemical approaches for controlling RTK signaling *in vivo*.

## Regulation of RTK activities with light

### Optogenetic control over RTK activities and downstream signaling

**Fig. 1:**
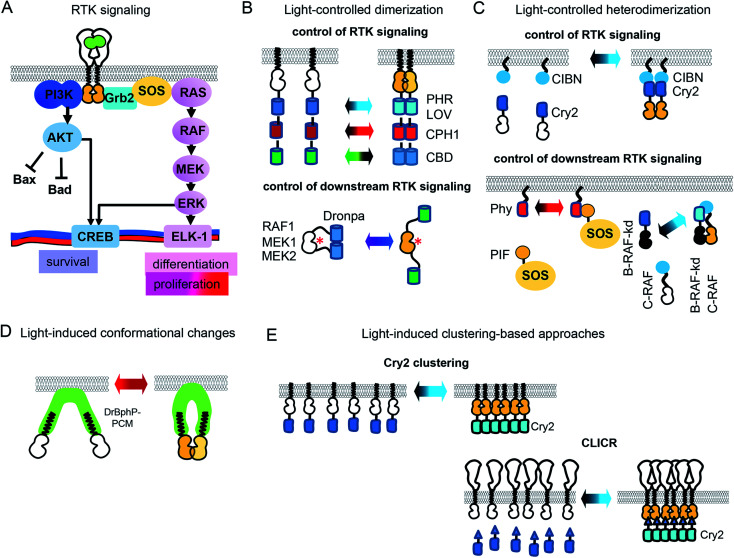
Design of opto-RTKs and ways to light-control RTK downstream signalling. (A) Activation of major RTK signalling pathways by growth factors. GF binding leads to the dimerization of the RTK and activation of the downstream signaling. (B) Light-controlled dimerization. Top: RTK intracellular domains are fused to photoreceptors, which dimerize upon action of light. This leads to dimerization and activation of RTKs. Bottom: Dimerization is used for the photocaging of the MEK catalytic center. (C) Light-controlled heterodimerization. Top: Heterodimerization for control of RTK signalling. Intracellular RTK domains are fused to cryptochrome 2 (Cry2). Illumination with blue light leads to the simultaneous translocation of Cry2-RTK to the PM and its activation. Bottom: Control of downstream RTK signalling. Light-controlled translocation of the SOS to the PM leads to activation of downstream ERK cascade starting from RAS. Heterodimerization of Cry2-B-RAF and CIBN–C-RAF-kd leads to the activation of the ERK cascade starting from MEK. (D) Light-induced conformational changes. RTK intracellular domains are attached to the photosensory core (PCM) of bacterial phytochrome of *D. radiodurans* (DrBphP). Upon action of near-infrared light DrBphP-PCM undergoes conformational changes, leading to RTK activation. (E) Light-induced clustering and CLICR. Top: RTK intracellular domains are fused to Cry2 photoreceptor. Light-induced clustering of Cry2 leads to the activation of opto-RTKs. Bottom: Endogenous RTK activation using CLICR. PLCγ-SH2-motif is fused to Cry2. Upon action of light SH2-Cry2 fusions cluster and interact with endogenous RTKs. Inactive RTK domains are shown in white while activated RTK domains are shown in orange.

In the simplified view of activation, RTK monomers dimerize after interaction with a GF, leading to trans-phosphorylation of RTK domains and subsequent activation of downstream signaling (Fig. 1A). However, the RTK activation process is more complex, and may depend on the reorganization of catalytic intracellular domains inside a preformed inactive dimer. There are significant differences in the reorganization mechanisms of various RTK families.^21^ Nonetheless, an induced dimerization suffices for the development of opto-RTKs constructs, as it has been demonstrated by a number of the engineered opto-RTK variants^10^,^22^ (Fig. 1B, C and Table 1). Other optogenetic principles, such as light-induced conformational changes and light-induced clustering, were also applied to the design opto-RTKs. A number of light-responsive protein modules are available for such engineering. They are able to homodimerize (Fig. 1B), heterodimerize (Fig. 1C), undergo conformational changes (Fig. 1D), or form clusters (Fig. 1E) upon action of light. Similar, light-responsive modules were used to control downstream RTK signaling, including kinases of the MAPK/ERK and PI3K/AKT pathways (Table 1).

**Table 1:** Optogenetic and optochemical tools controlling RTK activity

Optogenetic tools					
Photoreceptor	Light (nm)	Chromophore (its availability in mammalian cells)	Mechanism of light action	Applied to light-control	Ref.
**Cryptochrome 2 (Cry2) and photolyase homology domain of Cry2 (PHR)**					
PHR	Blue (∼455)	Flavin mononucleotide (available)	Homodimerization	TrkB, TrkA, TrkC	10
PHR			Homodimerization	FGFR1	10
Cry2olig (Cry2 E490G)			Clustering	EphB2	36
Cry2			CLICR	Non-specific activation of endogenous RTKs	14
Cry2-CIBN pair			Heterodimerization	TrkA	81

**LOV domains**					
VfAU1	Blue (∼455)	Flavin mononucleotide (available)	Homodimerization	mFGFR1, hEGFR, hRET	23

**Cyanobacterial phytochromes**					
CPH1	Far-red (∼630)	Phycocyanobilin (not available)	Homodimerization.	mFGFR1	13
	Near-infrared (∼780)		Dimer dissociation		
Phy–PIF pair	Far-red (∼630)		Heterodimerization	SOS	28
	Near-infrared (∼780)		Dissociation of the heterodimer		

**Cobalamin binding domains of CarH transcription factors**					
CBD	Green	Vitamin B12 (cobalamin)	Monomerization	mFGFR1	25

**Bacterial phytochromes**					
DrBphP-PCM	Far-red (∼630) and near-infrared (∼780)	Biliverdin (available)	Conformational changes	TrkA, TrkB	33

Optochemical systems					
Photocaged substance	Light (nm)	Photolabile group	Mechanism of light action	Applied to light-control	Ref.
**Incorporation of UAAs into protein active center by genetic code expansion**					
Tyr	UV	ONB	Photodeprotection	EgA1 (anti-EGFR) and 2Rs15d (anti-HER2) nanobodies	18
Tyr	UV	ONB		7D12 (anti-EGFR nanobody)	39
Lys	(450–780)	Coumarine derivatives		MEK1	17

**Semi-genetically encoded opto-RTKs and downstream signalling partners**					
Rapamycin	UV	ONB	Photocleavage	FKBP-FAK/FK506-FAK	40
Glutamate	UV	Azobenzene in BGAG_8_	Reversible uncaging of glutamate	VFTD-IR (LihIR)	15
Glutamate	UV			VFTD-c-Met	

**Photo-uncaging of RTK agonist**					
c-Met binding aptamer and its blockator	UV	ONB in linker	Photocleavage of the linker	c-Met	42
c-Met binding aptamer	Near-infrared (∼780)	Golden nanoparticles AuNRs	Phototermal uncaging (heating of AuNRs with 808 nm laser)	c-Met	7

**Chromophore-assisted light inactivation (CALI)**					
VEGFR2 binding peptoid^*a*^	Visible light	Ru(ii) (tris-bipyridil)^2+^	Visible light illumination leads to the release of the singlet oxygen in the vicinity of the RTK extracellular domain	VEGFR2	44

**Opto-PROTAC**					
PROTAC molecule, targeted to ALK	UV	NVOC	Photocleavage	ALK	20

^*a*^In CALI technique VEGFR2 peptoid, in principle, is not photocaged but rather is used to guide Ru(ii) (tris-bipyridil)^2+^ to VEGFR2 extracellular domain. Abbreviations: UV – ultraviolet; ONB – *o*-nitrobenzyl; BGAG_8_; FKBP – FK506 binding protein; FK506 – tacrolimus; FAK – focal adhesion kinase; PROTAC – proteolysis targeting chimera; NVOK – 6-nitroveratryloxycarbonyl; ALK – anaplastic lymphoma kinase; VEGFR2 – vascular endothelial growth factor receptor 2.

#### Light-controlled homodimerization

Light-controlled homodimerization was used for the development of the majority of opto-RTKs. In this engineering approach, the intracellular domain of the RTK monomer is deleted and a photoreceptor is attached to the intracellular domain N- or C-terminally. A membrane localization signal, such as a myristoylation peptide (Myr), is added to the N-terminus of the complete chimeric construct to target it to the plasma membrane (Fig. 1B).

The toolbox of available opto-RTKs includes blue, green, red and far-red/near-infrared light controlled RTKs (Fig. 1 and Table 1). Among blue-light controlled photoreceptors are light-oxygen-voltage (LOV) domains of *V. frigida* aureochrome 1 (VfAU1)^23^ and various derivatives of cryptochrome 2 (Cry2), including its photolyase homology domain (PHR).^10^ They dimerize upon action of blue light and use available in mammalian tissues flavin mononucleotide as a chromophore.^10^ These blue-light controlled opto-RTKs are widely used for *in vitro* and *in vivo* studies of RTK activity.^24^ Opto-RTKs controllable with green and red light are also available, but their major drawback is that they need addition of exogenous chromophores to cell culture medium. For example, a cobalamin-binding domain (CBD)-based opto-FGFR1 requires B12 vitamin as the chromophore.^25^ Far-red/near-infrared light controlled opto-RTKs were developed based on cyanobacterial phytochromes and bacterial phytochromes, for example cyanobacterial phytochrome 1 (CPH1) from *Synechocystis* was fused to an intracellular domain of fibroblast growth factor receptor FGFR1.^13^ CPH1 dimerizes upon exposure to far-red (∼630 nm) light and dissociates under near-infrared (∼780 nm) light (Fig. 1A). Far-red and near-infrared light exhibit lower phototoxicity and deeper penetrance into mammalian tissues, but CPH1-based opto-RTKs also need an external chromophore, such as phycocyanobilin.^13^

Light-controlled homodimerization can be also used to regulate downstream RTK signaling. Dimerization of a photoswitchable Dronpa (pdDronpa) protein was exploited for photocaging of catalytic centers of several kinases of the MAPK/ERK1/2 pathway (Fig. 1A). For example, photocaging of the catalytic centers of RAF1, MEK1 and MEK2 was achieved with pdDronpa that dimerizes upon exposure to UV light and monomerizes under blue light (Fig. 1B). Activation of the kinase was sterically inhibited by dimerization of pdDronpa and activated by monomerization.^26^,^27^

#### Light-controlled heterodimerization

Heterodimerization is a powerful way to activate RTKs and its downstream signaling. Several RTK signaling partners, such as guanine nucleotide exchange factor son of sevenless (SOS), are activated simply by translocating to the plasma membrane (Fig. 1A and B) and several heterodimerization systems can achieve this. In these, one of the monomers is attached to the membrane while the other is fused to a signaling protein (Fig. 1C). Illumination leads to the translocation of the relevant protein to the plasma membrane and activation of downstream signaling. For example, Phy–PIF heterodimerizing protein partners were used to light-control SOS. In this system Phy is attached to the membrane while SOS bears an N-terminal PIF fusion. Far-red light induces a Phy–PIF interaction, resulting in PIF-SOS translocation to the plasma membrane and activation of RAS-ERK signaling.^28^ In another example, a Cry2-CIBN heterodimerization by blue light can replace PI3K activation. Cry2 fused to the inositol 5-phosphatase domain of OCRL (5-ptase(OCRL)) translocates to the plasma membrane and induces the formation of phosphatidylinositol (3,4,5)-triphosphate (PIP3). Consequently, AKT translocates to the plasma membrane by binding to PIP3 through the PH domain.^29^

Moreover, light-induced heterodimerization allows studies of kinase heterodimers. Rapidly accelerated fibrosarcoma (RAF) kinases are represented by B-RAF and C-RAF isoforms. Excessive B-RAF signaling is oncogenic and is treated with RAF inhibitors. Paradoxically, low doses of B-RAF inhibitors induce stronger activation of downstream RAF signaling, including ERK activation.^30^,^31^ To reveal scaffolding role of inactive B-RAF, Chatelle *et al.* studied signaling of kinase dead B-RAF (B-RAF-kd)/C-RAF complex using optogenetics. To light-control RAF heterodimers, B-RAF-kd was fused to Cry2 while C-RAF was fused to CIBN. The Cry2-CIBN-mediated heterodimerization of C-RAF and B-RAF-kd activated RAF signaling stronger than C-RAF/C-RAF homodimerization, confirming role of B-RAF-kd as the activation scaffold of C-RAF (Fig. 1C).^31^

While the Cry2-CIBN light-induced interaction does not require exogenous chromophore, the Phy–PIF interaction needs addition of a phycocyanobilin chromophore, which complicates its use *in vivo*. However, the availability of a RpBphP1-QPAS1 heterodimerizing pair that relies on a biliverdin IXα chromophore, a product of heme catabolism, can overcome the requirement for an exogenous chromophore to control SOS and RAS signaling^32^

#### Light-controlled conformational changes

An important approach to developing far-red/near-infrared opto-RTK consists of fusing cytoplasmic RTK domains to a photosensory core module (PCM) of DrBphP bacterial phytochrome from *Deinococcus radiodurans*. DrBphP-PCM remains dimeric while undergoing substantial conformational changes when illuminated. Under far-red light, the distance between the C-termini of DrBphP-PCM can reach 3 nm (Fig. 1D), which is enough to prevent trans-phosphorylation of the kinase domains located at the C-terminus of DrBphP-PCM molecules. This approach was used to engineer optically-controlled TrkA and TrkB^33^ and optically-controlled EGFR and FGFR1.^34^ Bacterial phytochromes use biliverdin IXα that is readily available in mammalian tissues, and opto-RTKs engineered using bacterial phytochromes can function in stable cell lines and in transgenic animal models without external chromophores.^35^

#### Light-controlled clustering and CLICR

Light-induced dimerization, which is most frequently used in opto-RTK design, may be suboptimal for some receptors. For example, ephrin receptors form big oligomeric clusters on the cell surface, and efficient signaling is only possible after their oligomerization. Recently, a light-controlled EphB2 receptor was engineered by fusion with a Cry2 photoreceptor^36^ (Fig. 1E). Cry2 is the only photoreceptor that forms large oligomers upon illumination, which is currently used in optogenetic engineering.

All of the above-described opto-RTKs (Table 1) are chimeras of photoreceptors and the RTK kinase domain, and need to be expressed in cells heterogeneously. In contrast, a CLICR technique light-controls endogenous RTKs.^14^ In the CLICR approach, the N-terminal src-homology 2 (SH2) domain from PLCγ is fused N-terminally to Cry2. The SH2 domain interacts with cytoplasmic domains of endogenous RTKs. In darkness SH2-Cry2 weakly interacts with endogenous RTK, while blue light induces SH2-Cry2 clustering and increases its avidity to endogenous RTKs, leading to receptor oligomerization and activation (Fig. 1E). CLICR activates endogenous RTKs, such as FGFRs and PDGFR non-discriminately. The use of more specific N-terminal Cry2 fusions that recognize RTKs, such as mAbs against C-termini of RTKs could adapt CLICR to specific RTK targets.

### Optochemical means of controlling RTK activity

**Fig. 2:**
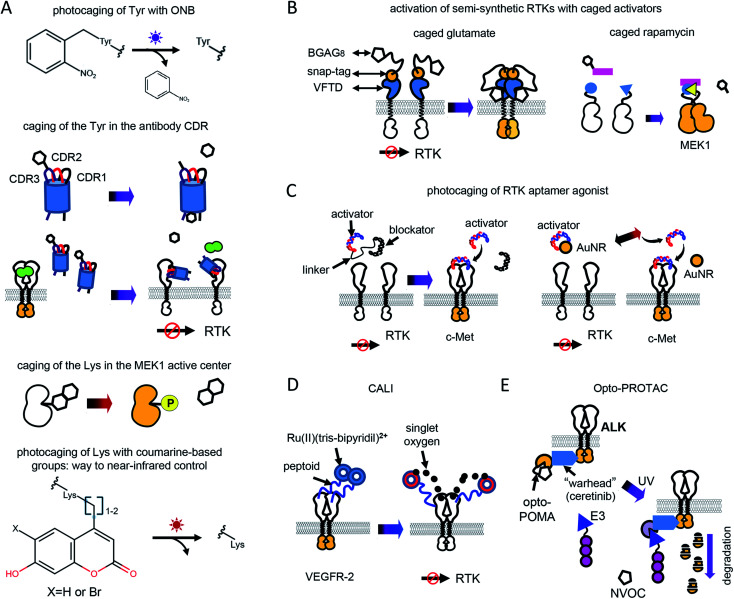
Optochemical means of controlling RTK activation. (A) Photocaging of amino acid residues. Top: Upon UV illumination ONB is photocleaved. Photodeprotection of Tyr in the CDR of nanobody enables binding of the extracellular EGFR domain and inhibition of its signalling. Bottom: Upon near-infrared illumination coumarine derivative is photocleaved. Photodeprotection of Lys in the active center of MEK1 results in kinase activation. (B) Development of semi-genetically encoded opto-RTKs. Left: Venus-flytrap (VFTD) based optochemical RTK activation. Ligand-binding domains of insulin receptor 1 (IR1) and c-Met are changed to VFTD of the GPCR mGluR2 with snap-tag. Labeling with BGAG8 makes VFTD-RTK chimeras photoactivatable. Upon illumination with UV light uncaged glutamate from BGAG8 binds VFTD which results in the VFTD-RTK chimera activation. Right: Photocaged rapamycin is able to induce dimerization of RTK domains only upon UV illumination. (C) DNA aptamer uncaging. Left: c-Met agonist (DNA aptamer) is linked to blocker aptamer with photocleavable linker. UV illumination leads to linker cleavage, agonist release and c-Met activation. Right: c-Met agonist (DNA aptamer) is conjugated to golden nanorods (AuNRs). Near-infrared illumination leads to heating of AuNRs, c-Met agonist release, endogenous c-Met activation. (D) CALI. VEGFR-2 binding peptoid is conjugated to Ru(ii) (tris-bipyridil)^2+^. Illumination leads to production of singlet oxygen, which inactivates VEGFR-2. (E) Opto-PROTAC approach. Ceretinib, specific to ALK (“warhead”) is connected to opto-POMA, UV illumination causes cleavage of NVOC group, interaction of POMA with E3 ligase and destruction of RTK intracellular domain.

Optochemical control of RTK signaling can be achieved by photolabile protecting groups (caging groups) linked to proteins, small molecule inhibitors, metal ions, aptamers and other substances (Fig. 2 and Table 1). Caging groups inhibit substance activity until light-induced uncaging. Optochemistry can regulate endogenous RTKs and opto-RTKs by a number of techniques, including photocaging of amino acids, such as tyrosines in the catalytic center of RTKs (Fig. 2A), use of photocaged small-molecule activators linked to RTKs (so called semi-genetically encoded opto-RTKs) (Fig. 2B), photocaging of RTK agonists (Fig. 2C), chromophore-assisted light inactivation (CALI) (Fig. 2D), and opto-PROTAC technique (Fig. 2E).

#### Photocaging of amino acids and anti-RTK antibodies

Photocaging of amino acids in RTKs and anti-RTK antibodies are reliable ways to block their function. The most frequently used for that photocaging is *o*-nitrobenzyl (ONB) group. Installation of a photocaging group can be performed through chemical modification of proteins *in vitro*, but it is more convenient to install relevant groups through the incorporation of unnatural amino acids, such as ONB-conjugated tyrosine (ONBY) (Fig. 2A). Illumination with UV light leads to deprotection of the tyrosine residues (Fig. 2A), resulting in activation of the relevant protein function, such as the ability to be phosphorylated or to bind to an antigen.

The incorporation of unnatural amino acids, which uses genetic code expansion through the amber codon suppression technique, involves engineering of orthogonal tRNA synthetases and their cognate rRNAs that are able to include unnatural amino acids in response to an amber stop codon (TAG). Initially, this method was used to incorporate tyrosines, lysines and serines conjugated with ONB. More recently, a tRNA synthetase capable of including coumarine-conjugated unnatural amino acids instead of TAG has been used.^37^

ONBY is widely used to modify active sites of proteins. Recently, light-activated nanobodies (termed photobodies) against the extracellular domains of EGFR and HER2 were developed by incorporating ONBY into complementarity determining regions (CDRs).^18^ For that, ONBY was inserted in the CDR3 loop of the anti-EGFR nanobody EgA1 (in place of Tyr119) and in the CDR1 loop of the anti-HER2 nanobody 2Rs15d (in place of Tyr37), using amber stop codon suppression technology.^38^ Upon illumination with UV light (∼350 nm) these photobodies bind extracellular domains of EGFR and HER2,^18^ whereas they remain inactive in darkness (Fig. 2A). Similarly, a light-activated nanobody against EGFR was developed by incorporating ONBY into the 7D12 anti-EGFR nanobody fragment.^39^

Photocleavage with UV light prevents application of the photocaged proteins in living animals. In contrast, use of coumarine as a photocaging group allows photodeprotection of amino acids with visible and near-infrared light. Recently, to light-control MEK1, a coumarine-caged lysine^17^ residue was installed into its active center (Fig. 2A). Likely, photocaging of lysine amino acids in CDRs of anti-RTK nanobodies with coumarine derivatives could shift their spectral sensitivity, enabling their usage *in vivo*.

#### Semi-genetically encoded opto-RTKs

In this approach, a RTK catalytic domain is fused to a protein, which dimerizes by binding to a small-molecule chemical dimerizer (CID), such as rapamycin or glutamate, that is photocaged. Illumination uncages the CID and promotes dimerization of semi-opto-RTKs. The most commonly used CID, rapamycin, binds to FK506-binding protein (FKBP) and to FKBP-rapamycin binding (FRB) domain of mTOR. Rapamycin caging can be achieved through installation of ONB at its C40 position. Illumination with UV light leads to photocleavage of the ONB group and restoration of CID activity (Fig. 2B). Recently, caged rapamycin was used to light-control focal adhesion kinase (FAK), which is a component of several RTK signaling pathways.^40^

Caged glutamate is used to light-control glutamate receptors. Using caged glutamate, semi-genetically encoded insulin (LihIR) and c-Met (LihMet) opto-RTKs have been developed.^15^ In these semi-opto-RTKs, the extracellular domains of both RTKs were swapped for the Venus flytrap domain (VFTD) of human metabotropic glutamate receptor type 2 (mGluR2) with N-terminal SNAP-tag, which is frequently used in design of glutamate biosensors^41^ (Fig. 2B). BGAG_8_, where BG is benzylguanidine for bioconjugation, A is an azobenzene photoswitch, and G is a glutamate head group, was used as a photolabile group that binds to SNAP. UV illumination (∼350 nm) causes glutamate from BGAG_8_ to uncage and bind VFTD. Upon glutamate binding, the C-terminal domains of VFTDs approach each other (Fig. 2B), causing trans-phosphorylation of intracellular RTK domains, which activates their signaling.^15^ In these semi-opto-RTKs the transmembrane domain is preserved and linked to extracellular VFTDs. In contrast to other opto-RTKs that preserve transmembrane domain and extracellular ligand-binding domain,^10^ LihIR and LihMet do not respond to endogenous ligands and, therefore, their light-activation is orthogonal to cell signaling.

#### Photo-uncaging of RTK agonist

Light activation of endogenous RTKs is also possible by photo-uncaging of their agonists, including DNA aptamers. DNA aptamers are oligonucleotides selected for specific recognition of certain targets using systematic evolution of ligands by exponential enrichment (SELEX) technique.^7^,^42^ A number of aptamers are available that bind extracellular domains of RTKs and activate them. Among them, a DNA aptamer that binds and activates hepatocyte growth factor (HGF) receptor c-Met, has been put under light control, using a photocontrolled DNA assembly approach.^42^

In this approach, three types of DNA oligonucleotides were designed to achieve light-control of c-Met. These were the DNA agonist itself and a blocker aptamer with a photocleavable linker. In darkness, DNA agonists were linked to the blocker aptamer through the photocleavable linker. Illumination with UV light led to the photocleavage of the linker and release of the aptamer (Fig. 2C).

Photothermal uncaging of the different c-Met activating aptamers was used to activate c-Met with near-infrared (808 nm) light.^7^ For that, DNA aptamer was conjugated with gold nanorods (AuNRs). Near-infrared illumination of conjugates locally heats AuNRs, releases aptamer, and activates endogenous c-Met (Fig. 2C). Notably, photothermal uncaging enables activation of endogenous c-Met in animal models.

#### Chromophore-assisted light inactivation (CALI)

The CALI approach relies on a production of reactive oxygen species (ROS) by the chromophore in the close proximity of the target protein.^16^,^43^ Singlet oxygen modifies various functional groups in proteins, but does not diffuse more than ∼8 nm from the generation point.^44^ There were attempts to inactivate RTKs by using conjugation of photosensitizing dyes to antibodies, however, their large size substantially decreased their efficiency.^16^,^44^ To overcome this spatial limitation, a small RTK binding peptide was used.^44^ In this approach the chemically stabilized peptide analogue GU40C (peptoid), which binds VEGFR2, was conjugated to derivatives of Ru(ii) (tris-bipyridil)^2+^. Ru(ii) (tris-bipyridil)^2+^ is one of the most efficient producers of singlet oxygen, activated with UV light. Derivatives of Ru(ii) (tris-bipyridil)^2+^ were made to produce singlet oxygen upon illumination with visible light.^44^ When conjugated to the VEGFR-2 binding peptoid, these Ru(ii)-containing compounds generate singlet oxygen and inactivate VEGFR2 (Fig. 2D). This technique could be adapted for inactivation of oncogenic VEGR2 signaling.

#### Opto-PROTAC

Controlled inhibition of endogenous RTK signaling could be used for treating certain cancers.^5^ The extracellular domains of RTKs are a therapeutic target for mAbs while intracellular RTK domains are a target for small molecule inhibitors. RTKs frequently develop resistance to both types of therapy due to oncogenic mutations. This can lead to constitutive dimerization of RTKs or to single-amino acid activating mutations in the vicinity of a catalytic center. In contrast to mAbs-based or inhibitor-based therapies, a recently developed technique called (PROteolysis Targeting Chimera) PROTAC^45^ can destroy whole RTK molecules because its specificity is not limited to extracellular domains or the catalytic center, which may undergo single-amino acid oncogenic mutations.^20^,^45^

PROTAC consists of two parts: a target-specific molecule and an E3-ligase interacting molecule. Interaction of PROTAC with the E3 ligase tags the target for degradation. This technique was successfully used for treating several inhibitor-resistant cancers. However, non-optically controlled PROTAC demonstrated some cytotoxicity *in vivo* by degrading target proteins in normal tissues.^20^

To develop an optically-controlled PROTAC (opto-PROTAC), which targets anaplastic lymphoma kinase (ALK), Liu *et al.*^20^ used a light-insensitive version of the ALK-degrading (dALK) PROTAC that was composed of ALK inhibitor ceretinib linked to pomalidomide (POMA) using a short linker.^45^ In the light-insensitive dALK, a light-insensitive POMA interacts with the E3 ubiquitin ligase CUL4-RBX-DDB1-CRBN (CRL4(CRBN)), and ceretinib binds ALK (Fig. 2E). A light-sensitive POMA (opto-POMA) was engineered by installing 4,5-dimethoxy-2-nitrobenzyloxycarbonyl (NVOC) group on it.^20^ The resulting opto-POMA was further used to design an optically controlled dALK (opto-dALK). In the opto-dALK, UV light caused photocleavage of the NVOC group and interaction of E3 ligase with POMA, resulting in degradation of the ALK intracellular domains. Similarly to photobodies, opto-PROTAC could be used to destroy oncogenic RTKs in target tissues only with a focused light beam.

## Opto-RTKs and light-control of endogenous RTKs in cancer research

Overactivation of RTKs is a hallmark of cells undergoing oncogenic transformation. It can be caused by a local increase in growth factor concentrations or oncogenic mutations that leads to constitutive RTK activation.^23^ Oncogenic diseases are frequently treated with mAbs that block RTK interactions with GFs or with small molecule inhibitors that inhibit RTK phosphorylation. However, many tumors develop resistance to both types of therapies. Moreover, indiscriminate inhibition of RTK signaling may cause side-effects in normal tissues thereby making tight control of RTK inhibition or destruction desirable.

As an example, EGFR is overexpressed in a number of solid tumors, and is targeted with anti-EGFR antibodies and small-molecule inhibitors.^5^,^46^ However, EGFR signaling plays a central role in skin biology,^47^ and high doses of anti-EGFR mAbs and small molecule inhibitors cause adverse effects on skin, including papulopustular skin rashes, dryness, and infections.^47^

**Fig. 3:**
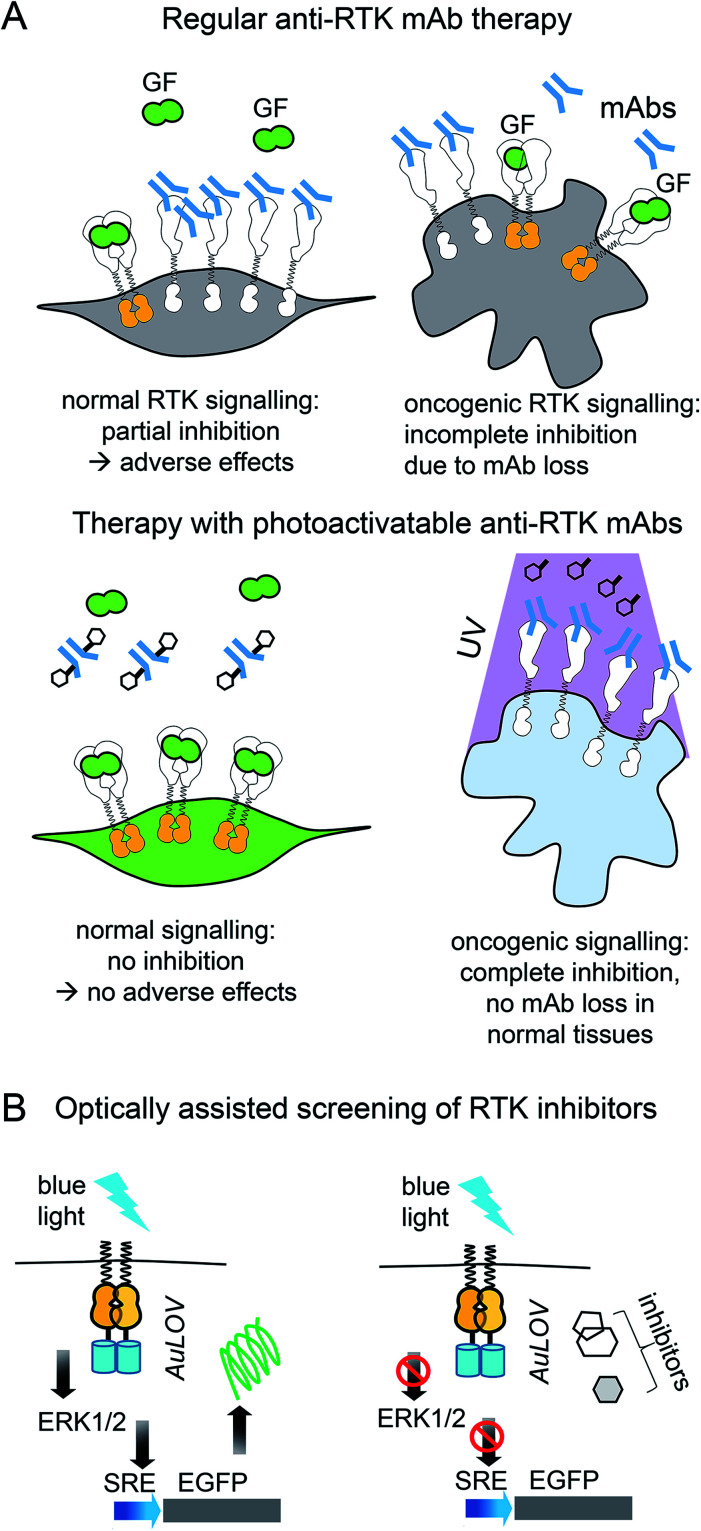
Cancer therapy with photoactivatable antibodies and all-optical screening of RTK inhibitors. (A) Photobodies in cancer therapy. Comparison of the regular anti-RTK mAbs therapy and therapy with photobodies. Top: Therapy with regular anti-RTK mAbs: injected antibodies interact with RTKs expressed both on the surface of normal (left) and oncogenic (right) cells. This results in the reduction of the concentration of mAbs, reaching the tumor and adverse effects in normal tissues, due to partial inhibition of RTK signaling in normal cells.^46^ Bottom: Therapy with photobodies. Injected antibodies interact only with RTKs expressed on the surface of tumour cells after illumination of tumour with UV light (right). There is no mAbs loss in normal tissues and there is no inhibition of normal RTK signaling. There are no adverse effects either.^90^. (B) Screening of RTK inhibitors using opto-RTKs. Cells expressing opto-RTKs (opto-FGFR1, opto-EGFR or opto-ROS1) and a MAPK/ERK pathway-responsive GFP reporter (SRE-GFP) are activated with light, and pathway activation is detected using GFP reporter. Cells are treated with prospective small molecule RTK inhibitors. If the substance inhibits RTK signalling, then GFP signal reporting MAPK/ERK signal activation is absent. The approach requires not contact to the cells, solution exchange, reagent addition with exception of addition of prospective RTK inhibitors.^20^

Light-control of anti-RTK mAbs could help to avoid non-specific inhibition of RTK signaling in normal tissues (Fig. 3A). Similarly, light-controlled destruction of RTKs, for example with opto-PROTAC, could obliterate RTKs only in tumor but not in normal tissues. Whereas light-controlled destruction and inhibition of RTKs holds great promise for cancer therapy, opto-RTKs and other optically-controlled kinases can be useful for delineating the different branches of downstream RTK signaling (Fig. 1A) in cancer development and in drug screening.

Light-controlled activation of C-RAF–B-RAF-kd heterodimers demonstrated the role of B-RAF-kd as an activation scaffold of C-Raf.^30^ It has also enabled the elucidation of why some B-RAF inhibitors used in cancer therapy act as paradoxical C-RAF activators. For screening of inhibitors, opto-RTKs allow all-optical assays in which cells are co-transfected with an opto-RTK for activation in one spectral range and with an ERK1/2-activity fluorescent reporter providing a readout in another spectral range (Fig. 3B).^23^ This assay allowed to identify a novel hROS1 small-molecule inhibitor Tivozanib (AV-951). Tivozanib was tested in an all-optical assay for its ability to block hROS1, mFGFR1 and hEGFR, and was shown to inhibit hROS1 signaling only.^23^

Photocontrollable anti-RTK antibodies and RTK destruction with opto-PROTAC should be useful in cancer therapy. For now, only UV-controlled photobodies and opto-PROTAC are available. Success in engineering of optogenetic and optochemical tools sensitive to deeply-penetrating non-phototoxic near-infrared light portends the development of similar constructs operating in the near-infrared spectral range.

It is noteworthy that excessive RTK activation may cause not only cancer but a number of other diseases reviewed elsewhere.^48^ The examples include various craniosynostosis syndromes caused by FGFR constitutive activation^48^ and abnormal retinal vascularization caused by excessive VEGFR2 signaling.^49^ Therapy and studies of pathogenesis of such diseases using light-controlled antibodies and opto-RTKs could be considered.

## Opto-RTKs in diseases linked to insufficient RTK signaling

Insufficient expression of GFs and other RTK ligands leads to a number of disorders (Table 2). A notable example is diabetes mellitus, which is caused by insufficient insulin production or insensitivity of tissues to insulin.^2^ Other examples are Laron syndrome, a growth delay linked to insufficient serum level of insulin-like-growth factor 1 (IGF1),^50^ and neurodegenerative diseases accompanied by a deficiency in nerve growth factors.^6^ Treatment of such diseases involves replacement of RTK ligands. However, such replacement therapy is often inefficient because the target tissues may deregulate RTK function, as it happens in diabetes mellitus type II and in Alzheimer's disease in which the TrkA expression in cholinergic neurons is decreased. Heterogeneous expression of opto-RTKs in the affected tissues could become an alternative to replacement therapies in diabetes mellitus, neurodegeneration and regenerative medicine.

**Table 2:** Diseases linked to insufficient RTK signalling^*a*^

Disease	RTK involved	GF replacement therapy or other therapy	Ref.
Diabetes mellitus type I	Insulin receptor 1 (IR1)	Insulin	2
Diabetes mellitus type II	Insulin receptor 1 (IR1)	Metformin	82
Growth delay, dwarfism	Insulin-like growth factor receptor 1 (IGF1)	Insulin-like growth factor (somatomedin)	50
Neurodegeneration	TrkA	NGF	6
Diabetic foot ulcers	EGFR, PDGFR	EGF, PDGF	4 and 83
Coronary artery disease	c-Met	HGF	84

^*a*^Abbreviations: TrkA – tropomyosin receptor kinase A; NGF – neurotrophic growth factor; EGF – epidermal growth factor; PDGF – platelet derived growth factor; EGFR – EGF receptor; PDGFR – PDGF receptor; c-Met – tyrosine-protein kinase Met; HGF – hepatocyte growth factor.

### Opto-RTKs for therapy of neurodegeneration

**Fig. 4:**
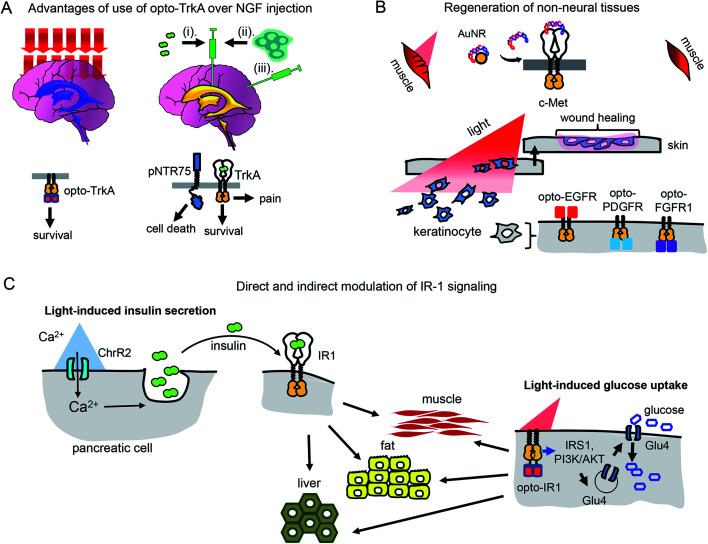
Perspective of optical control of RTK activity in humans and model animals. (A) Advantages of usage of opto-TrkA over NGF injection. Non-invasive activation of opto-TrkA in cholinergic neurons in patients with Alzheimer's disease. Left: Activation of opto-TrkA in the forebrain can be performed non-invasively. Light activates only opto-TrkA, promoting survival of cholinergic neurons. Right: (i) NGF delivered through injection in the choroid plexus diffuses to peripheral nervous system promoting adverse effects. (ii) NGF produced by genetically modified autologous patient fibroblasts injected in the nucleus basalis of Meynert activates not only TrkA, but “death receptor” p75NTR. (B) Regeneration of non-neural tissues. Top: Example of repair of muscle damage in rodents by activation of endogenous c-Met with light.^7^ The similar approach involving delivery of photo-caged c-Met activator can be applied for treatment of muscle damage in humans. Bottom: Wound repair in skin or cornea by light-activation of opto-EGFR, opto-PDGFR and opto-FGFR1 can be performed by delivery of opto-RTKs into keratinocytes and their migration towards the wound. (C) Treatment of diabetes mellitus with help of optical manipulation of insulin secretion and opto-RTKs. *Ex vivo*: autologous pancreatic cells are transformed with ChR2 and injected back to the patient. Illumination with light causes insulin release, which activates IR1 in key insulin-sensitive tissues. *In vivo*: optically controlled IR1 is delivered to key insulin-sensitive tissues. Light activation of opto-IR1 induces activation of PI3K/Akt signalling cascade, translocation of the Glu4 glucose transporter to the cell membrane and glucose uptake.

Use of opto-RTKs to treat neurodegeneration in the CNS seems especially attractive because of immune privilege of the CNS. The first clinical trials of NGF in the therapy of mild Alzheimer's cases demonstrated that intra-cerebral infusion of NGF leads to adverse effects, including back pain, because of NGF diffusion into the peripheral nervous system. However, a clinical trial of Ceregene, which involved injection of autologous primary fibroblasts transduced with adeno-associated virus serotype 2 (AAV2) encoding human NGF (CERE-110) to the nucleus basalis of Meynert, demonstrated that injection of NGF-overexpressing cells was safe and well-tolerated. Nevertheless, it did not affect clinical outcomes of biomarkers in Alzheimer's disease,^51^ probably because of the TrkA downregulation in cholinergic neurons that has been reported in several Alzheimer's disease studies.^6^ Cholinergic neurons express two types of NGF receptors, including TrkA that induces cell survival and pNTR75 that induces cell death. Opto-RTK-based treatment of Alzheimer's disease could involve delivery of opto-TrkA to the nucleus basalis of Meynert followed by activation with near-infrared light, so that only pro-survival signaling is activated^6^ (Fig. 4A).

Unlike treatment with NGF and NGF-producing fibroblasts, use of opto-TrkA for Alzheimer's therapy could resolve several issues. First, only activation of opto-TrkA expressed in forebrain cholinergic neurons will be performed, thereby bypassing activation of pNTR75 or TrkA receptors in the peripheral nervous system. Second, activation of opto-TrkA with light could be applied not constitutively but in a dose-dependent manner that can be adjusted during the course of treatment. Third, such treatment will fully rely on heterogeneous opto-TrkA expression and will not depend on low expression of endogenous TrkA receptors in the early stages of Alzheimer's disease. Lastly, because of the targeted activation of opto-TrkA in the forebrain, specifically in nucleus basalis of Meynert, this approach will not result in adverse effects observed in the NGF therapy.

### Opto-RTKs for non-neural tissue regeneration and tissue engineering

Humans and other mammals, as opposed to some lower vertebrates, have low regeneration capacity. They are not able to regenerate full limbs, like axolotl salamander. Their capacity to regenerate skin and muscle is also limited and often results in scar formation that prevents full recovery of skin function.^52^ Regeneration of wounded tissues and wounded skin depends on the action of a number of factors including RTK ligands, such as EGF (stimulates proliferation and migration of keratinocytes and increases tensile strength of new skin), PDGF (acts as a chemoattractant for mesenchymal cells), FGF (stimulates proliferation, migration and angiogenesis in injured skin), and VEGF (initiates angiogenesis and stimulates proliferation and migration of endothelial cells).^9^ Insufficient production of these GFs may diminish regenerative capacity of wounded tissues (Table 2).

PDGF became the first recombinant GF approved by Food and Drug Administration (FDA) for topical administration in the therapy of diabetic foot ulcers.^53^ Modern dermal replacement scaffolds derived primarily from extracellular matrix proteins, such as collagen and elastin, are used to treat severe skin wounds and burns.^54^ It has been demonstrated that EGF and another EGF family member, neuregulin-1 (NRG1), are able to promote proliferation and migration of fibroblasts and keratinocytes towards such artificial scaffolds.^54^ In these cases, opto-RTKs, such as opto-EGFR could be delivered to fibroblasts and keratinocytes in the wound site using AAV particles. Gradient application of activating light could induce directional migration of the opto-RTK expressing cells towards the wound site, improving healing (Fig. 4B).

Optochemical approaches for wound healing and regeneration of liver and muscles are also conceivable. The above-described optochemical caging of c-Met agonist with AuNRs nanorods was used to treat muscle damage in rodents. The near-infrared light-induced release of DNA aptamers activated the c-Met receptor in the damaged skeletal muscle and enhanced its healing^7^ (Fig. 4B).

### Diabetes mellitus

There are two types of diabetes mellitus. The cause of diabetes type I is death of insulin-producing pancreatic β-cells (Table 2). Insulin is a ligand of the insulin receptor 1 (IR1), which is responsible for glucose uptake from the bloodstream. An insulin replacement therapy, most commonly the administration of recombinant human insulin,^2^ is used to treat type I diabetes. In type II diabetes, insulin is still produced by pancreatic cells but its interaction with IR1 fails to induce glucose uptake, leading to insulin resistance.^2^

Recently, several optogenetic constructs allowing modulation of IR1 signaling were developed. They exploit the possibility of secondary messengers to induce insulin secretion by autologous pancreatic β-cells or MIN6 cells and consist of photoactivatable adenylyl cyclase (PAC) from *Beggiatoa* that induces cAMP synthesis^55^ and light-activated cation channel channelrhodopsin 2 (ChR2) that induces Ca^2+^ influx^56^ (Fig. 4C). The insulin-producing cells bearing such optogenetic tools could be transplanted into animals after encapsulation in polymer capsules consisting of semi-permeable layers enabling diffusion of nutrients but protecting them from the immune system.^55^,^56^ In the future, this strategy could advance treatment of diabetes type I in humans.^57^,^58^

However, for the therapy of diabetes mellitus type II in which tissues have lost sensitivity to insulin, a direct induction of glucose uptake with a light-controllable opto-IR1 in major insulin target tissues, such as liver, muscles and adipose tissue, could be required (Fig. 4C).

## Application of opto-RTK technologies in animal models

In animal models of human diseases, opto-RTKs could be used to exploit the role of RTK signaling in development^59^ and behavior.^24^ In behavioral studies by Tan *et al.*,^60^ TrkB was ablated postnatally from the majority of corticolimbic GABAergic interneurons. These TrkB cKO mice exhibited intact motor coordination and movement but had enhanced dominance over other mice in a group-housed setting. The authors then transduced the TrkB-deficient GABAergic interneurons with the eArch3.0 outward proton pump that hyperpolarizes (inhibits) cells when activated by green-yellow light. The optogenetic suppression of firing of these neurons completely reversed the dominance behaviour in TrkB cKO mice. These results suggested a role for BDNF/TrkB signalling in inhibitory synaptic modulation and social behaviour. However, to study directly BDNF/TrkB signalling, one of the available opto-TrkB constructs could be used instead of eArch3.0.

A large number of animal models of RTK-related human diseases, a selection of which is presented in Table 3, is available. Opto-RTKs could be easily implemented in these models. The obvious targets are animal models of diabetes mellitus type I and II, amyotrophic lateral sclerosis, and Alzheimer's disease.

**Table 3:** Animal models of human diseases related to RTK signalling^*a*^

Animal model	RTK ligand or RTK modified	Genetic modification	Phenotype	Ref.
**Diabetes**				
Ins2(Akita)	Misfolding of insulin	Single a.a. substitution in insulin 2 gene causing protein misfolding	Male mice heterozygous for this mutation have progressive loss of beta-cell function, decreased pancreatic beta-cell density, significant hyperglycemia at 4 weeks of age	85
BIRKO	IR1 knockout	Knockout of IR1 in pancreatic β-cells	Impaired insulin response to glucose challenge, impaired glucose tolerance, high insulin level	86
NIRCO	IR1 knockout	Neuronal tissue specific IR1 knockout	Elevated body weight, white adipose tissue, serum triglycerides, circulating leptin (changes mostly pronounces in females)	86

**Growth delay and dwarfism**				
Igf1^–/–^	IGF1	Deletion of IGF1 gene	Growth restriction (30% of adult size)	87
Igf1^m/m^		MIDI	Growth restriction, reduced femoral length	87
Igf1^+/–^		Haploinsufficiency	Growth restriction (70% of adult size), reduced femoral length	87
Igf1r^+/–^	IGF1R	Haploinsufficiency	Growth restriction (90% of adult size)	

**Neurodegeneration**				
Alzheimer's disease (AD11)	NGF	NGF antibody is expressed in brain and neutralizes mature NGF *versus* unprocessed proNGF	Progressive neurodegeneration which resembles many features of AD; atrophy and loss of cholinergic neurons in a brain region; accumulation of phosphorylated tau filaments in 2 month-old AD11 mice in entorhinal region; spreading with age to other cortical and hippocampal areas; accumulation of insoluble tau in aged AD	3
VEGF ΔHRE	VEGF	Deletion of the hypoxia-response element in *Vegf* promoter	Late-onset motor dysfunction; reduced hypoxic Vegf expression in spinal cord	88

**Degeneration of non-neural tissues**				
Epidermal-EGFR deleted mice	EGFR	EGFR ablation in skin	Development of skin lesions after one-week of age	89

^*a*^Abbreviations: IR1 – insulin receptor 1; BIRCO – pancreatic beta-cell specific insulin receptor knockout mouse; MIRCO – mouse with muscle-specific insulin knockout; IGF1 – insulin-like growth factor; IGF1R – IGF1 receptor; NGF – neurotrophic growth factor; VEGF – vascular endothelial growth factor; ΔHRE – deletion in hypoxia response element; EGFR – receptor of epidermal growth factor.

## Challenges of implementing opto-RTKs in curing diseases

Application of opto-RTKs in translational studies may face several challenges, similar to previously implemented gene therapies (*e.g.* FDA-approved Luxturna gene therapy for retinitis pigmentosa). The major challenges are delivery of opto-RTKs to their action sites with viral and non-viral vectors, immune rejection in non-privileged tissues, and ways of delivery of light to deep-seated organs.

### Delivery of opto-RTKs to target cells, tissues and organs

**Fig. 5:**
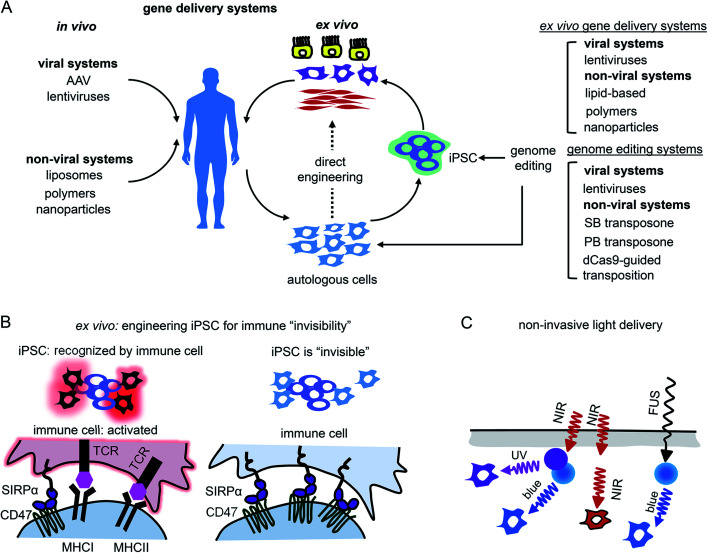
Challenges of using of opto-RTKs in translational research and possible solutions. (A) Delivery of opto-RTKs to target tissues. *In vivo*: viral and non-viral vectors are directly injected into the patient. *Ex vivo*: autologous patient cells are directly transfected with opto-RTKs or differentiated into pluripotent stem cells (iPSCs), differentiated into specialized cells and injected back into the patient. (B) Overcoming host immune response. Autologous host cells are engineered to be invisible to host immune system by inactivation of major histocompatibility complex (MHC) and over-expression of CD47. Cells subsequently are transduced with opto-RTKs and are injected back into the patient. (C) Delivery of light towards RTK action sites. Use of up-conversion light-absorbing nanoparticles allows to activate opto-RTKs in deep organs with near-infrared light and focused ultrasound.

Gene therapy can be performed *in vivo* when a vector is injected into a patient or *ex vivo* when autologous cells (*e.g.*, hematopoietic stem cells or photoreceptor retinal cell precursors) are genetically transformed and transplanted back into a patient (Fig. 5A).

The application of opto-RTKs to gene therapy requires safe and efficient gene delivery systems. These systems should provide long-term expression of opto-RTK in the target cells or tissues and have a large DNA packaging capacity. Opto-RTKs are usually encoded by long genetic sequences, sometimes more than 5 kb as in the case of the HER family.^34^

*In vivo* gene therapies rely on the use of viral vectors. Among them, recombinant adeno-associated viral vectors (rAAVs) were used in clinical trials including delivery of light-sensitive proteins.^61^,^62^ Key features of rAAVs for their *in vivo* use are safety and ability to infect various types of mammalian cells, including non-dividing cells, such as neurons.^63^ In contrast to wild-type AAV particles, rAAVs are replication-deficient and not able to replicate even in the presence of a helper virus. rAAVs are used in modern gene therapy approaches. Moreover, several gene therapy clinical trials of optogenetic tools, such as delivery of ChR2 conducted by Allergan (#02556736) and delivery of red-light activatable cation channel ChrimsonR conducted by GenSight Biologics (#03326336), use rAAVs for transgene delivery to the retina.^64^ Two major drawbacks of rAAVs are their limited packaging capacity (∼5 kb) and inability to integrate into a host genome. While the latter property enhances a rAAV safety, it also results in a loss of transgene copies in dividing cells, thereby requiring new rAAV injections to maintain transgene expression. This latter limitation makes rAAVs inapplicable to *ex vivo* gene therapy.^62^

Lentiviral vectors (lentivectors) is an alternative for opto-RTK delivery in target tissues for both *in vivo* and *ex vivo* gene therapy. Similarly to rAAVs, third generation lentivectors are engineered to be replication-deficient.^63^,^65^ As opposed to rAAVs, lentivectors are able to integrate into the host genome, which is important for constitutive transgene expression moreover, they possess substantial packaging capacity of ∼8 kb.^65^ The major drawback of lentivectors is that their integration into a host genome may cause insertional mutagenesis. However, the development of lentivectors capable of site-specific integration should eliminate this risk.^63^ Because of their integration in a host genome lentivectors are used in *ex vivo* gene therapies, including FDA-approved ones, such as Kymriah that is based on the lentiviral modification of autologous CAR T-cells.^65^

Non-viral gene transfer by means of plasmid vectors can be used for *ex vivo* gene therapy^66^ but typically leads to transient transgene expression in target cells and does not ensure gene integration in the host genome. Efficiency of gene incorporation in the genome *ex vivo* can be improved using non-viral transposon-based systems (Fig. 5A). These gene delivery systems require cotransfection of transposon DNA with a transposase as an expression plasmid or as mRNA. The gene insert is recognized and excised from the plasmid by transposase, which then inserts the transgene into the host genome. The most commonly used transposon systems are Sleeping Beauty and PiggyBac.^67^,^68^ Importantly for heterogeneous expression of opto-RTKs, a cargo capacity of the latter system is larger (up to 200 kb) than that of Sleeping Beauty (up to 11 kb). Moreover, efficiency of the genomic insertion by Sleeping Beauty system substantially drops for genes exceeding 2 kb, making it less preferable for the large opto-RTK constructs. For therapeutic applications it is also important to cotransfect transposase as mRNA to avoid its accidental insertion in the host genome and, consequent, genomic instability or oncogenesis.^67^

Whereas Sleeping Beauty and PiggyBac transposon systems insert genes in a host genome in non-predictable locations, a site-specific gene integration can be performed using an RNA-guided transposition. Several such techniques are available, and the following are used the most often. First, bacterial Tn7-like transposons have coopted nuclease-deficient CRISPR-Cas systems to catalyze RNA-guided integration of mobile genetic elements into the genome.^69^ Integration of donor DNA occurs in one of two possible orientations at a fixed distance downstream of target DNA sequences and can accommodate variable-length gene inserts. Involving a fully programmable RNA-guided integrase, it enables highly specific genome-wide DNA insertion across dozens of unique target sites. Second, similar principle allows the integration of genes into the human genome with the SP transposon and catalytically inactive Cas9 (dCas9) directed by a single guide RNA (sgRNA) against human *Alu* retrotransposon.^70^

We anticipate that both *in vivo* and *ex vivo* gene delivery strategies could be implemented for transferring of opto-RTK constructs to target tissues in therapy.

### Suppressing rejection of opto-RTKs by the immune system

The other challenge for opto-RTKs use in the clinic is their possible rejection by the host immune system. Opto-RTKs are based on bacterial photoreceptors, which when expressed in mammalian tissues represent a target for the immune system and, consequently, lead to photoreceptor-induced immunogenicity.^71^ The CNS has immune privilege and is able to tolerate the expression of foreign proteins without eliciting an immune response. This enables usage of optogenetic tools for vision restoration in humans. Similarly, this may allow implementation of opto-RTKs for the treatment of neurodegeneration.

To reduce immune responses in tissues that are not immune-privileged several strategies could be exploited. First is the use of immunosuppressive drugs as tacrolimus.^71^ Second is encapsulation of therapeutic cells bearing optogenetic constructs with materials shielding them from action by the immune system.^72^,^73^ Third, genetic modifications of therapeutic cells could be used, especially for *ex vivo* gene therapies. It has been shown that inactivation of both major histocompatibility complexes (MHC) class I and class II with simultaneous overexpression of CD47 rendered mouse and human pluripotent stem cells “invisible” to the immune system^74^ (Fig. 5B). The two last strategies seem to be the most feasible for application to optogenetic tools, like opto-RTKs, both *ex vivo* and *in vivo* too.

### Ways of light delivery to opto-RTK expression tissues

Light delivery needs to be optimized for optogenetic tools that are expressed in deep tissues. Shorter wavelengths penetrate mammalian tissues less efficiently than longer wavelengths, yet most opto-RTKs are activated with blue light. Non-specific activation of opto-RTKs with ambient light should be considered as well.

In animal models light delivery is performed using an implanted optical fiber, and this approach cannot be employed in humans. The problem of light delivery in human therapy can be solved in several ways (Fig. 5C). First, the most straightforward one is to use opto-RTKs activated with near-infrared light that penetrates mammalian tissues substantially better than visible light.^35^,^75^ Second, lanthanide nanoparticles allow conversion of near-infrared light from the activating light-source into shorter wavelength light. Consequently, this triggers optogenetic tools sensitive to blue, green and yellow light. In theory, by proper selection of dopants, lanthanide-doped nanoparticles can be made to emit light at wavelengths that cover almost the entire visible spectrum. Third, mechanoluminescent nanoparticles that respond to focused ultrasound (FUS) can be used.^76^ Mechanoluminescence refers to light emission from various organic and inorganic materials in response to mechanical stimuli, such as friction, tension, fracture and compression.^77^ Mechanoluminescent nanoparticles can be delivered into the circulation using intravenous injection and turned on with ultrasound focused at the target tissue to repetitively emit 470 nm light thereby activating common opto-RTKs. Ultrasound penetrates tissues deeper than near-infrared light but exhibits lower spatial precision.

## Future outlook

A vast array of optogenetic and optochemical technologies allows activation and inhibition of RTK signalling by light. While precise inhibition of endogenous RTK activity with light is needed to treat tumors, which are limited in size and location, RTK activation is equally valuable for the treatment of diseases linked to RTK insufficiency. Optogenetic and optochemical methods of RTK control are successfully used in cultured cells and can also be applied in animal models of diseases (Table 3). However, how safely and how efficiently they can be transferred to the clinic and what are advantages and disadvantages of either technique?

Optochemical techniques have the advantage over optogenetic approaches of being less immunogenic. However, the majority of optochemical methods of RTK regulation, including photoactivatable therapeutic anti-RTK antibodies rely on UV light that is highly cytotoxic. Therefore, one of the future directions in the development of optochemical approaches of RTK control is to shift their responsiveness towards the near-infrared part of the spectrum. Substantial efforts have already been made using coumarine to design photolabile groups sensitive to near-infrared light.^37^ Similarly to the near-infrared-switchable MEK1,^17^ conjugating amino acids in the CDR regions of antibodies to photolabile red-shifted groups should result in far-red and near-infrared activatable photobodies. However, optochemical methods of RTK control could also be considered more invasive than optogenetic techniques. For example, they may require multiple injections of recombinant photobodies into the bloodstream to achieve therapeutic effect.

Optogenetic means of controlling RTK activity rely on a large number of photoreceptors that sense light in various parts of light spectrum, and the first near-infrared opto-RTKs have recently been developed using bacterial phytochrome as a scaffold.^33^ One future direction will be the development of near-infrared activatable antibodies inhibiting RTK signalling for precision cancer therapy. This could be achieved with a nanobody light-induced complementation approach reported for blue light-activatable heterodimerizers.^78^ Protein engineering efforts will be required to replace the blue optogenetic domains with near-infrared light-controllable heterodimerizers, such as BphP1-QPAS1.^32^

Although optogenetic techniques can control degradation of proteins fused to photoreceptors, these approaches do not allow degradation of endogenous proteins. Given the vast selection of optogenetically controlled heterodimerizers, a fully genetically encoded analogue of opto-PROTAC could also be engineered. A non-optogenetically controlled PROTAC analogue already exists and consists of nanobody against SPOP, an adaptor protein of cullin-RING E3 ubiquitin ligase, and a second nanobody that recognizes a protein target.^79^ Both nanobodies could be fused to available optogenetic near-infrared heterodimerizers,^32^ enabling light-activatable degradation of endogenous proteins, like RTKs or their downstream counterparts.^80^

Implementation of opto-RTKs should be beneficial in various translational studies, including type II diabetes and different types of cancer. Various light-activation modes applied to opto-IR1 expressing diabetic mice could aid in understanding of the nature of insulin resistance and help to optimize schedules of insulin injection in diabetes type II patients. One of the biggest challenges of using opto-RTKs in the clinic is their potential immunogenicity. The successful application of optogenetic tools for vision restoration in immunologically privileged tissues should encourage use of opto-RTKs in treating neurodegeneration in the CNS. The development of strategies for safe and precise gene delivery and suppression of immune response in genetically modified tissues will result in the use of opto-RTKs in non-neural tissue engineering.

A number of GF replacement therapies fail to improve conditions of patients. We anticipate that in the foreseeable future, optogenetic and optochemical technologies of RTK regulation will become available as alternative therapies for diseases linked to insufficient ligand production and impaired RTK signaling, as well as will provide efficient RTK inhibition and destruction with photoactivatable therapeutic antibodies and RTK inhibitors, giving the patients hope.

## Conflicts of interest

The authors declare no conflict of interests.
